# Analysis of macrosegregation formation and columnar-to-equiaxed transition during solidification of Al-4 wt.%Cu ingot using a 5-phase model

**DOI:** 10.1016/j.jcrysgro.2014.07.039

**Published:** 2015-05-01

**Authors:** M. Ahmadein, M. Wu, A. Ludwig

**Affiliations:** a Chair for Modeling and Simulation of Metallurgical Processes, University of Leoben, Leoben, Austria; b Production Engineering and Mechanical Design Department, Faculty of Engineering, Tanta University, Tanta, Egypt; c Christian-Doppler Laboratory for Advanced Process Simulation of Solidification & Melting, University of Leoben, Austria

**Keywords:** Macrosegregation, Sedimentation, CET, As-cast structure, Solidification, Ingot casting, Modeling

## Abstract

A 5-phase mixed columnar-equiaxed solidification model was recently introduced to predict the as-cast structure, and a series of laboratory experiments were performed previously to verify the model. The focus of the current work is to analyze the formation of macrosegregation, which accompanies the formation of the as-cast structure including the columnar-to-equiaxed transition (CET). The as-cast structure and macrosegregation map of a cylindrical Al-4 wt.% Cu ingot poured at 800 °C are used as a reference to validate the calculations. Good agreement between the calculations and the experiment regarding both the macrosegregation and CET is achieved. Thermal-solutal convection and equiaxed crystal sedimentation in such ingot are verified to be key mechanisms governing the formation of macrosegregation. The competitive equiaxed/columnar growth, the soft and hard blocking mechanisms predominate the CET. The numerical study of grid sensitivity indicates that the global segregation pattern and CET are not significantly affected by grid size; however, some fine details of the segregation map which are predicted by fine grid (~0.5 mm) are smeared or locally averaged by the coarse grid (~2 mm). Such details were also not resolved in the measurement. Future investigations are demanding.

## Highlights

• This article provided a numerical study for the formation of the as-cast structure, including the columnar-to-equiaxed transition, and the macrosegregation pattern. Good agreement to experiment was achieved. Accordingly, detailed analysis for the sequence of solidification was conducted.
• The origin of equiaxed and columnar crystals and the positive and negative macrosegregation is explained and discussed.
• This contribution has a scientific and engineering importance. It improves the understanding of the solidification phenomenon and clarifies the interaction between melt flow, equiaxed crystal nucleation and sedimentation, and the solidification structure, which is experimentally impossible to trace. Engineers and material developers can control the solidification structure and avoid the formation of macrosegregation based on the detailed analysis provided in this work.

## Nomenclature

$c_{l},c_{e},c_{c}$: concentration of l-, e-, c- phases
$c_{d}^{c},c_{s}^{c}$: concentration of interdendritc melt and solid in *c*-phase
$c_{d}^{e},c_{s}^{e}$: concentration of interdendritc melt and solid in *e*-phase
$c_{0}$: initial alloy composition
$c_{E}$: eutectic concentration
$c_{mix}$: mixture concentration
$c_{s}^{⁎},c_{l}^{⁎}$: equilibrium solid and liquid concentration at solid/liquid interface
$C_{lc},C_{le}$: species exchange rate between phases, $\mathrm{kg}\;m^{-3}\;s^{-1}$
$C_{ds}^{e},C_{ds}^{c}$: species exchange rate at liquid-solid interface, $\mathrm{kg}\;m^{-3}\;s^{-1}$
$D_{l},D_{s}$: liquid and solid diffusion coefficients, $m^{2}\;s^{-1}$
$f_{l},f_{e},f_{c}$: volume fraction of phases
$h_{l},h_{e},h_{c}$: enthalpy, $J\;{\mathrm{kg}}^{-1}$
k: partitioning coefficient of solute
$k_{l},k_{e},k_{c}$: thermal conductivity, $W\;m^{-1}\;K^{-1}$
m: slope of liquidus in phase diagram, K
$M_{lc},M_{le}$: interphase mass transfer rate, $\mathrm{kg}\;m^{-3}\;s^{-1}$
$M_{ds}^{e},M_{ds}^{c}$: mass transfer rate at liquid-solid interface, $\mathrm{kg}\;m^{-3}\;s^{-1}$
$M_{tip^{׳}}^{c}$: mass transfer rate due to growth of primary c-dendrite tip, $\mathrm{kg}\;m^{-3}\;s^{-1}$
n: equiaxed grain number density, $m^{-3}$
$n_{c}$: number density of columnar dendrites, $m^{-3}$
N: equiaxed grain production rate, $m^{-3}\;s^{-1}$
p: pressure, $N\;m^{-2}$
$Q_{el}^{d},Q_{cl}^{d}$, $Q_{ce}^{d}$: interphase heat exchange rate, $J\;m^{-3}\;s^{-1}$
$Q_{el}^{p},Q_{cl}^{p},$$Q_{ce}^{p}$: energy exchange rate due to latent heat, $J\;m^{-3}\;s^{-1}$
$R^{c}$: radius of volume equivalent cylinder for columnar trunk envelope, m
$R^{e}$: radius of volume equivalent sphere for equiaxed grain envelope, m
$S_{env,M}^{c}$: surface concentration of equiaxed grain, $m^{-1}$
$S_{env,M}^{e}$: surface concentration of equiaxed grain, $m^{-1}$
$S_{s}^{c},S_{s}^{e}$: s-d interface concentration in columnar and equiaxed phase, $m^{-1}$
$T_{f}$: melting point of pure metal (Al), K
$T_{l},T_{e},T_{c}$: temperature, K
ΔT: constitutional undercooling, K
${\vec{u}}_{l},{\vec{u}}_{e},{\vec{u}}_{c}$: velocity vectors of phases, $m\;s^{-1}$
${\vec{U}}_{le}^{d},\;{\vec{U}}_{lc}^{d}$: momentum exchange rate due to interphase drag forces, $\mathrm{kg}\;m^{-2}\;s^{-2}$
${\vec{U}}_{le}^{p},\;{\vec{U}}_{lc}^{p}$: momentum exchange rate due to phase change, $\mathrm{kg}\;m^{-2}\;s^{-2}$
$v_{cell}^{c}$: growth velocity of cellular trunk, $m\;s^{-1}$
$v_{env}^{c}$: growth velocity of columnar trunk envelope
$v_{env,M}^{c}$: growth velocity of volume-equivalent cylinder of columnar trunk envelope, $m\;s^{-1}$
$v_{tip\prime }^{c},$ growth velocities of primary and secondary
$v_{tip\prime \prime }^{c}$: dendrite arms of c-phase, $m\;s^{-1}$
$v_{env}^{e}$: growth velocity of equiaxed grain, $m\;s^{-1}$
$v_{glob}^{e}$: growth velocity of globular *e*-grain, $m\;s^{-1}$
$v_{env,\;M}^{e}$: growth velocity of *e*-grain (volume equivalent of spherical envelope), $m\;s^{-1}$
$v_{tip\prime }^{e}$: growth velocity of equiaxed dendrite tip, $m\;s^{-1}$
$v_{sd}^{c},v_{sd}^{e}$: growth velocities of s-d interface in the columnar and equiaxed phases, $m\;s^{-1}$
$\alpha_{d}^{c},\;\alpha_{s}^{c}$: volume fraction of interdendritic liquid and solid in the columnar dendrite trunk
$\alpha_{d}^{e},\;\alpha_{s}^{e}$: volume fraction of interdendritic liquid and solid in the equiaxed dendrite
$\lambda_{1},\;\lambda_{2}$: primary and secondary dendrite arm spacings, m
$\rho_{l},\;\rho_{e},\;\rho_{c}$: density of phases, $\mathrm{kg}\;m^{-3}$

**Subscripts**
l,e,c: liquid, equiaxed, and columnar hydrodynamic phases
d: interdendritic liquid
*s*: dendritic solid

## 1 Introduction

Macrosegregation is a phenomenon associated with solidification of engineering castings. In contrast to microsegregation, it occurs over larger length scale of 1 cm–1 m. Homogenization heat treatment and diffusion mechanisms cannot remove macrosegregation due to its large length scale [1]. Macrosegregation can be very harmful for the material either in service or during subsequent processing.

The formation of macrosegregation during solidification cannot be traced experimentally. Only the end result can be detected by post-mortem examination of the cast structure. Several numerical models were developed to predict macrosegregation and improve the understanding of its origin. The first treatment of inverse macrosegregation was introduced by Scheil [2]. Flemings and Nereo suggested one dimensional model to predict segregation based on a local solute redistribution equation [3]. Two-phase (liquid and solid) model based on continuum formulation was proposed [4–7]. The thermal and solutal buoyancy and the shrinkage-induced fluid flow were taken into account. The model succeeded to predict the inverse segregation close to the bottom of the ingot. Beckermann et al. introduced an equiaxed dendritic model which takes into account the grain nucleation and the combined motion of liquid and equiaxed crystal [8–10]. The model demonstrated promising results for segregation of Al-4 wt.% Cu alloy and further development was recommended [11]. As an extension to the previous efforts and the classical model proposed by Hunt [12], several models were developed to combine the columnar solidification with the equiaxed to predict the columnar-to-equiaxed transition [13–16]. A non-dendritic mixed columnar/equiaxed model was applied to predict macrosegregation formed in large steel ingot [17]. However, discrepancy between predictions and experiment was obtained and attributed to mainly to the over-simplification of the dendritic morphology. Very recently Wu et al. extended the previous works by introducing a 5-phase volume-averaging model [18–21]. The new model tackled the dendritic morphology of crystals. This model considered additional interdendritic liquid phases that influence the formation of macrostructure and macrosegregation. The 5-phase model was verified to be able to quantitatively predict the as-cast structure of Al-alloy ingots [22,23] and the solidification of the ammonium chloride transparent solution [24].

The current work aims at improving the understanding of solidification phenomenon and the origin of the associated macrosegregation map. The aforementioned 5-phase model is used to reproduce the as-cast structure and the macrosegregation map, which were determined experimentally on an Al-4 wt.% Cu ingot.

## 2 Numerical aspects

### 2.1 The model

**Fig. 1:**
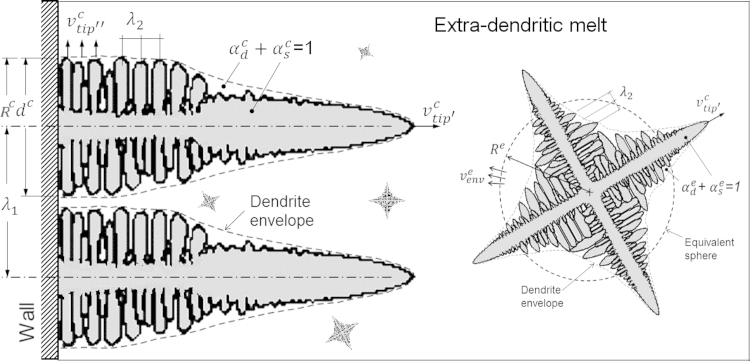
Schematic illustration of the phase mixture showing the dendrite envelope, the volume-equivalent sphere and *e*- for *c*- phases, respectively, and the inter- and extra-dendritic melt.

The 5-phase model comprises three hydrodynamic phases: liquid melt, equiaxed crystals, and columnar crystals. They are quantified by their respective volume fractions $f_{l}$, $f_{e}$ and $f_{c}$. Globular and dendritic growth of equiaxed crystals and cellular and dendritic growth of columnar grains are considered in the model. In case of dendritic growth, two additional phase regions exist within each of the equiaxed and the columnar crystal envelopes as shown in Fig. 1: the solid dendrites with corresponding solid fractions $\alpha_{s}^{e}$ and $\alpha_{s}^{c}$, and interdendritic melt with corresponding volume fractions $\alpha_{d}^{e}$ and $\alpha_{d}^{e}$ inside crystal envelopes. Consequently, the system encompasses five ‘thermodynamic’ phases: (1) the solid dendrite, (2) the interdendritic melt within the equiaxed grain, (3) the solid dendrite, (4) interdendritic melt within the columnar crystal envelope and (5) the extradendritic melt. The corresponding averaged volume fractions are $f_{s}^{e}$, $f_{d}^{e}$, $f_{s}^{c}$, $f_{d}^{c}$ and $f_{l}$ referring to total volume and they characterize by corresponding solute concentration$\;c_{s}^{e}$, $c_{d}^{e}$, $c_{s}^{c}$, $c_{d}^{c}$ and $c_{l}$. The envelope acts as a fictitious crystal boundary which connects the dendrite tips to separate the interdendritic melt from the extradendritic melt. The envelope shape is further simplified by a volume-equivalent sphere for the equiaxed crystal and a volume-equivalent cylinder for the columnar grain with corresponding radii $R^{e}$ and $R^{c}$ as shown in Fig. 1.

All conservation equations are summarized in Table 1. The liquid and equiaxed crystals move freely under the effect of thermosolutal buoyancy forces. They exchange the momentum as given in Eqs. (11) and (12) in Table 1, whereas the columnar dendrites which are assumed to stick to the mold walls. The motion of equiaxed crystal is blocked in the equiaxed region when $f_{e}>0.637$ or when it is captured by the columnar trunks in the mixed zone if $f_{c}>0.2$. The advancement of the columnar tip front can be prevented either mechanically (hard blocking) when $f_{e}$ ahead of the columnar primary tip front exceeds 0.49 [12] or thermodynamically (soft blocking) [13] due to the solutal interaction with the equiaxed crystals in the vicinity of the columnar tip front, when liquid melt at the columnar tip is enriched with solute to the extent that the constitutional undercooling vanishes. At this moment, columnar-to-equiaxed (CET) transition occurs.

The growth of the grain envelope and the solidification of the interdendritic melt are treated differently. The growth of the envelopes is determined by dendrite growth kinetics using the Kurz-Giovanola-Trivedi (KGT) model for the growth of columnar primary dendrite tips and the Lipton-Glicksman-Kurz (LGK) model for the growth of columnar secondary dendrite tips and equiaxed primary dendrite tips [25,26]. The solidification of the interdendritic melt is driven by the supersaturation of the interdendritic melt and governed by the diffusion in the interdendritic melt region. A heterogeneous nucleation law with three experimentally determined fitting parameters ($\Delta T_{M}$, $\Delta T_{\sigma }$, $n_{max}$) [27–29] is used to calculate the nucleation source term for the equiaxed number density (Eq. (16)). The source terms of the conservation equations of mass and species are provided in Table 1. The mixture concentration, $c_{mix}$, is calculated as the weighted average of the concentrations of the l-, e- and c-phases as given in Eq. (26). The regions with $c_{mix}>c_{0}$ encounter a positive macrosegregation and those with $c_{mix}<c_{0}$ encounter a negative macrosegregation. Further details of the model can be referred to [18–21].

### 2.2 Simulation settings

**Fig. 2:**
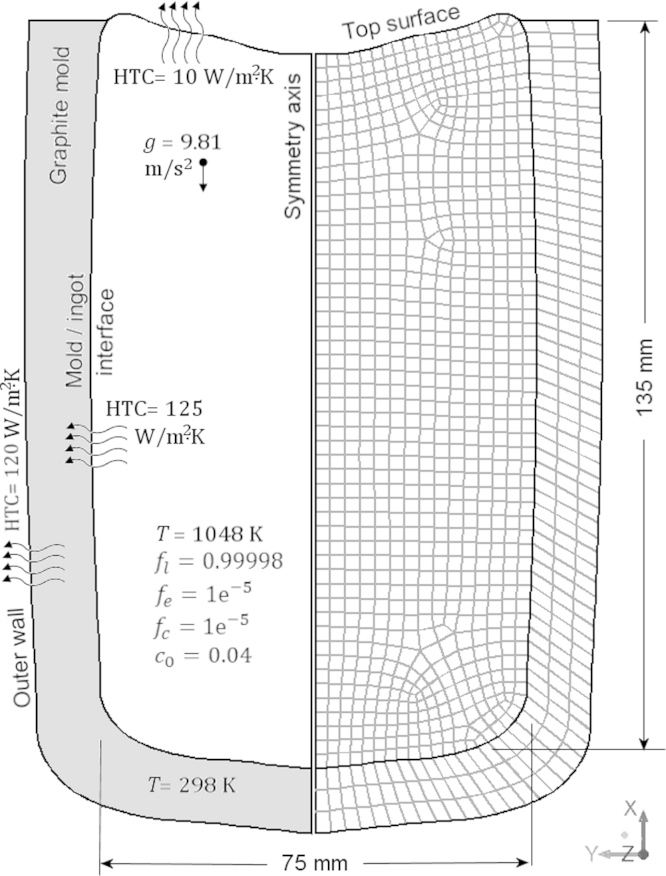
2D-axisymmetric grid (~2 mm), boundary and initial conditions of the ingot casting.

The solidification of the cylindrical Al-4.0 wt.%Cu ingot (75 mm diameter×135 mm height) in a graphite mold is calculated using a 2D axisymmetrical grid. The heat transfer through the mold was also taken into account. Initial and boundary conditions are provided in Fig. 2. The temperature of mold and melt were set initially at 298 K and 1048 K respectively. The thermophysical properties of the alloy and the necessary modeling parameters are listed in Table 2. The influence of grid size on macrosegregation and CET was investigated. In addition to the coarse (1030 elements ×~2 mm) grid, simulation was repeated using a fine (3714 elements ×~1 mm) and a very fine grid (14028 elements ×~0.5 mm).

The conservation equations given in Table 1 and the corresponding source terms were implemented within an Eulerian multiphase code. Using the CFD software package, ANSYS-Fluent version 14.5.0, the equations are solved sequentially at each time step with implicit linearization based on the control volume method. Converged solution in parallel computation was achieved using a time step of $1.0\times 10^{-3}$ to $5.0\times 10^{-5}$s, depending on the grid size.

## 3 Simulation results

### 3.1 Solidification sequence

**Fig. 3:**
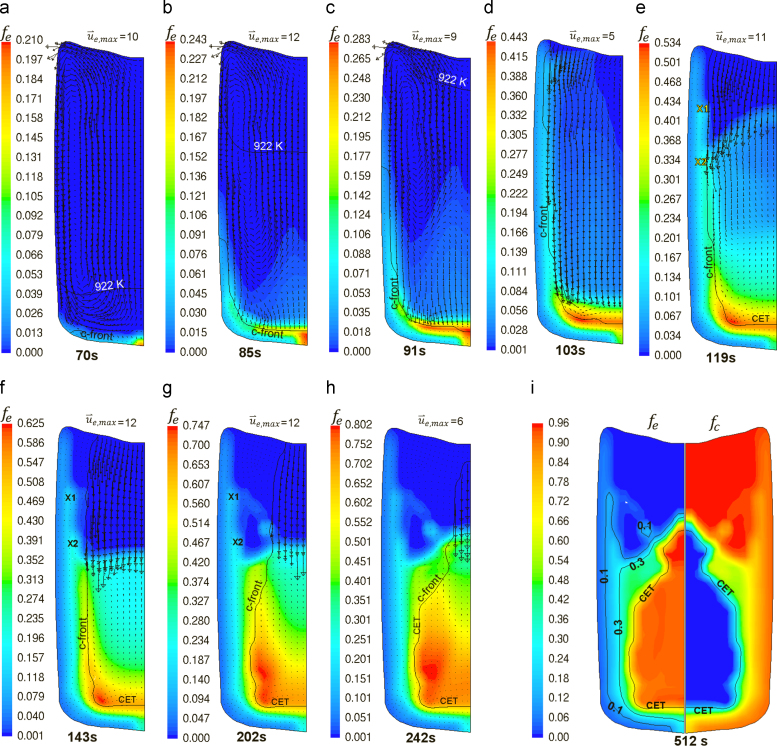
Solidification sequence of ingot (a-h) showing the contour of $f_{e}$ at different times with ${\vec{u}}_{e}$ ($\mathrm{mm}\;s^{-1}$) superimposed and (i) final as-cast structure ($f_{e}$, and $f_{c}$ contours and CET).

The development of volume fraction of equiaxed phase and the corresponding velocity vector (superimposed) are tracked over the solidification period as shown in Fig. 3. The columnar front (c-front) is fictitious line connecting the columnar primary dendrite tips. The advancement of this line resembles the growth of the columnar phase region. The isotherm of liquidus temperature shown in Fig. 3(a–c) is just an indicator to the temperature development within the ingot. The melt between the liquidus isoline and the c-front is supercooled.

In less than 100 s, the temperature of the entire ingot sinks to below the liquidus temperature. Competitive growth of *e*- and *c*-phases starts at the bottom of the mold after 70 s. Sedimentation of equiaxed crystals accompanied by melt convection is very effective at all stages of solidification. At very beginning (70 s, Fig. 3a), the liquid close to the mold side wall sinks down while that at the ingot centre floats up due to thermal buoyancy. The tiny equiaxed solid (the model minimum with $f_{e}$=10^−5^) in bulk obeys the dominant flow of the liquid. Further cooling (85 s, Fig. 3b) increases both $f_{c}$ and $f_{e}$ at the wall. The falling liquid stream at the side wall carries the solid crystals towards the mold bottom. The larger crystals disobey the liquid and settle in the mold bottom whereas the smaller crystals are carried up by the liquid due to the drag force. The columnar zone continues to thicken, especially at the bottom corner, and to expand towards the top. Equiaxed crystals behind the advancing c-front are captured by the columnar dendrites. The columnar skin of the ingot reached the top after 103 s (Fig. 3d) while equiaxed crystals are showering in the bulk, which causes stacking of equiaxed solid ahead of the growing c-front at the bottom. The velocity vectors, ${\vec{u}}_{e}$, at the top are deflected due to the drag of liquid at the region. The deflection of ${\vec{u}}_{e}$ is much obvious in Fig. 3e at the markers X1 and X2. Higher $f_{e}$ is formed in the markers’ zone. Further sedimentation and growth of equiaxed crystals increase $f_{e}$ ahead of the c-front at the ingot bottom to surpass the hard blocking limit (0.49). At this point, the so-called columnar-to-equiaxed transition (CET) occurs. Accordingly, the c-front at this region is frozen till the end of solidification, whereas the c-front at other regions is allowed to advance. In the late stage, no equiaxed crystals nucleate at the ingot top any more (Fig. 3e–h) due to the reduced undercooling. Nevertheless, columnar phase is progressively advancing and converging towards the centerline at the ingot top. Simultaneously, the existing equiaxed crystals are gradually settling to stack at the bottom. As a result, the advancement of the c-front in the bottom region is gradually blocked from the bottom-up. Finally, the as-cast structure is formed as shown in (Fig. 3i). The bottom half exhibits an equiaxed core and mixed peripherals with high $f_{c}$. The top half is mainly columnar with tiny amount of equiaxed grains captured by the columnar dendrites close to the mold walls.

**Fig. 4:**
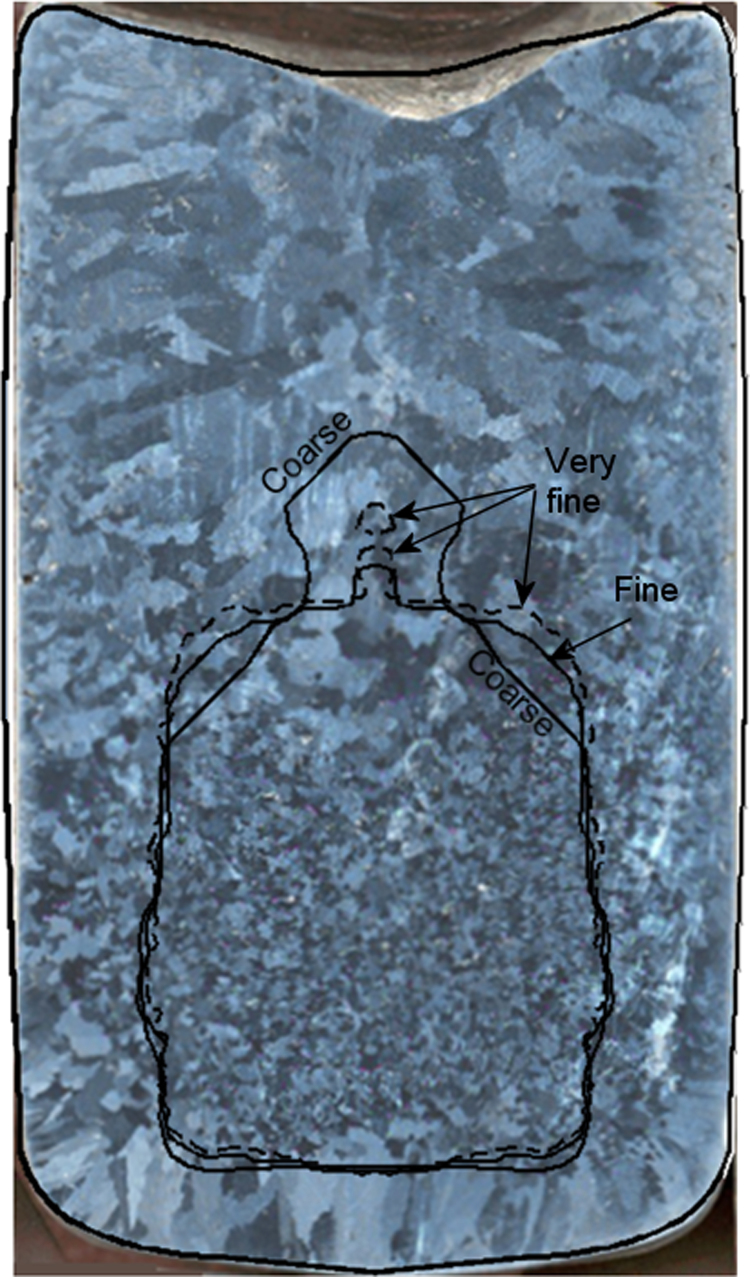
Calculated CET using different grids superimposed on the as-cast structure of casting experiment.

The calculated CET line from the above case is superimposed on the as-cast structure from the experiment as shown in Fig. 4. The calculation results from different grids are also overlaid. Several points of agreement between the calculated (Fig. 3i) and the real as-cast structure can be summarized as follows: (1) the upper ingot half is fully columnar, (2) the core of lower ingot half is primarily equiaxed, (3) the upper boundary of the equiaxed core (the swelling region) is extended upward, (4) the peripherals of the lower ingot half exhibits a mixed columnar/equiaxed structure and (5) the mixed zone close to the mold wall is extended towards the top.

### 3.2 Macrosegregation

**Fig. 5:**
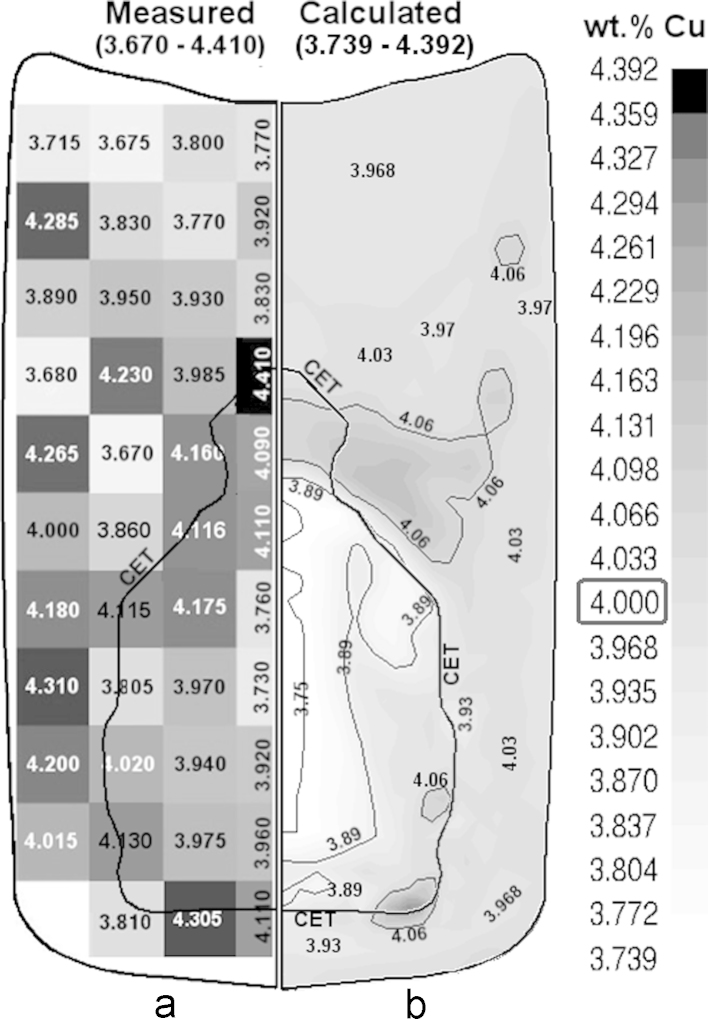
(a) Measured solute concentration (from spectrometer analysis with 5–8 mm spark diameter) compared to (b) the calculated mixture concentration, $c_{mix}$, using ~2 mm grid.

The cast ingot is cut and prepared for element analysis using X-ray spectroscopy (spark analysis). A grey scale map of the distribution of the measured solute concentration is constructed as shown in Fig. 5a. The predicted $c_{mix}$ contour using the coarse grid (~2 mm) is shown in Fig. 5b. By comparing both experimental and calculation results, the key features of the segregation map agree with each other: (1) the measured concentration, $c_{mix}$, falls in a range between 3.67 and 4.41 wt.% Cu compared to a predicted range 3.739~4.392 wt.% Cu; (2) the top of the upper ingot half has negative macrosegregation; (3) the equiaxed core of the ingot exhibits an extreme negative macrosegregation; (4) the mixed columnar/equiaxed zone at top boundary of the CET line exhibits positive macrosegregation; (5) the bottom boundary of CET contains dispersed regions of positive macrosegregation, (6) the mixed columnar/equiaxed structure between CET and mold wall is positively segregated and (7) several discrete sites of positive and negative macrosegregation exist in the upper part of the ingot. Detailed analysis of the segregation map formation mechanisms is made in Section 4.

### 3.3 Grid sensitivity

The calculation of the as-cast seems to be less sensitive to the grid size. As shown in Fig. 4, the predicted CET lines using different grid fit each other, especially in the lower part. They agree with experiment as well. Nevertheless, the configuration of the upper boundary of CET deviates slightly, depending on grid size. A conical top CET boundary with equiaxed ‘swelling’ is predicted using the coarse grid whereas the finer grids exhibit a flatter top CET boundary with smaller ‘swelling’. This can be attributed to the volume averaging approach used for formulating the conservation equations. As explained in Fig. 3, the equiaxed crystals settle down, stack in the equiaxed ‘basin’ and simultaneously grow. The columnar front is blocked and CET occurs if the condition $f_{e}>0.49$ is fulfilled in the volume elements ahead of the c-front elements. For the finer grids, this condition fulfilled at the top earlier than for the coarse grid. The averaged $f_{e}$ in coarse grid is quite less than 0.49 which promotes further advancement and delays CET, forming a conical CET at the top. In addition, the incremental front advancement (consult [20] for front tracking) is slightly grid dependent. This means that the c-front ‘jumps’ according to the grid size from one element to the next, as far as the c-front tip is sufficiently undercooled. Smoother tip advancement occurs in the case of the finer grids. Consequently, an equiaxed swelling is formed in the case of the coarse grid due to chocking of the swell region at its base caused by the stepwise advancement of the c-front. Thus, equiaxed crystals are captured within the equiaxed swelling and prevented from further settling. The extradendritic liquid is enriched in solute rejected from the growing solid. The c-front advancement at the region of the equiaxed swelling is then constitutionally (due to solute enrichment of liquid) and mechanically (when $f_{e}>0.49$) blocked.

**Fig. 6:**
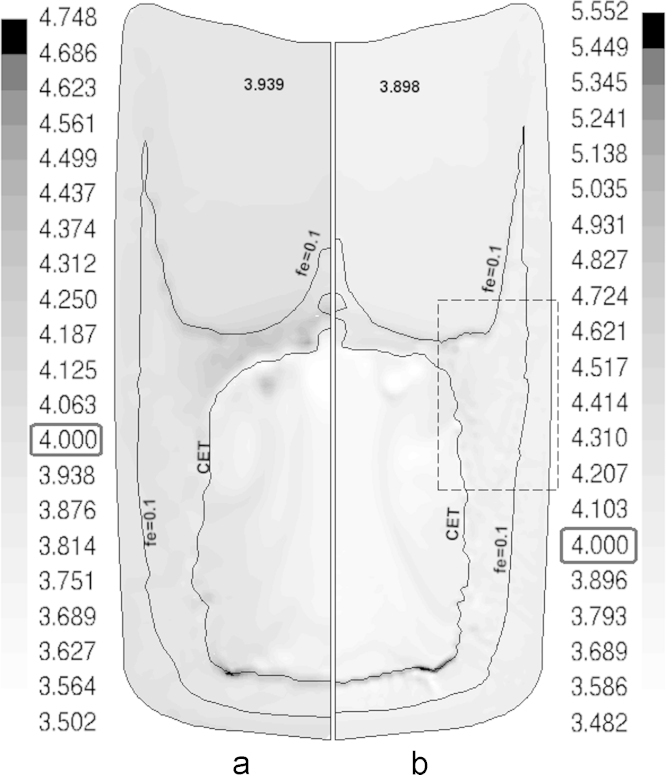
Calculated solute concentration using (a) ~1 mm and (b) ~0.5 mm grids.

The calculated $c_{mix}$ results using finer grids are shown in Fig. 6. The finer grids (1 and 0.5 mm) exhibit a global macrosegregation distribution similar to that of the coarse (2 mm) grid (Fig. 5b): negative at the upper part and at the core of the ingot and positive close to the top and bottom of CET. However, the positively segregated islands formed at the peripherals of the top half are much dispersed in the case of fine grids. Furthermore, the distribution of $c_{mix}$ range (maximum/minimum) is stretched by refining the grid and significantly deviates from the measurements and the predictions of the coarse grid (Fig. 5). This can be attributed to the concept of volume averaging. The localized high/low $c_{mix}$ appears in the fine grid is smeared or averaged by using coarser volume elements. In practice, eutectic (the solute-rich phase) forms normally at the grain boundaries and in the interdendritic regions. The length scale of such solute-rich regions can be in the order of secondary dendrite arm spacing, depending on the eutectic amount and the gain size. For instance, the measurements shown in Fig. 5a represent the average wt.%Cu in the spark area of the X-ray spectrometer. The diameter of the spark in the current case ranges between 5 and 8 mm. Obviously, using a more confined spark will reduce the sampling area and correspondingly will provide more localized concentration measurements that differ from those of wide spark, which is analogously expected to occur in the simulation if a fine grid is used.

## 4 Analysis and discussion

### 4.1 Segregation in columnar zone

**Fig. 7:**
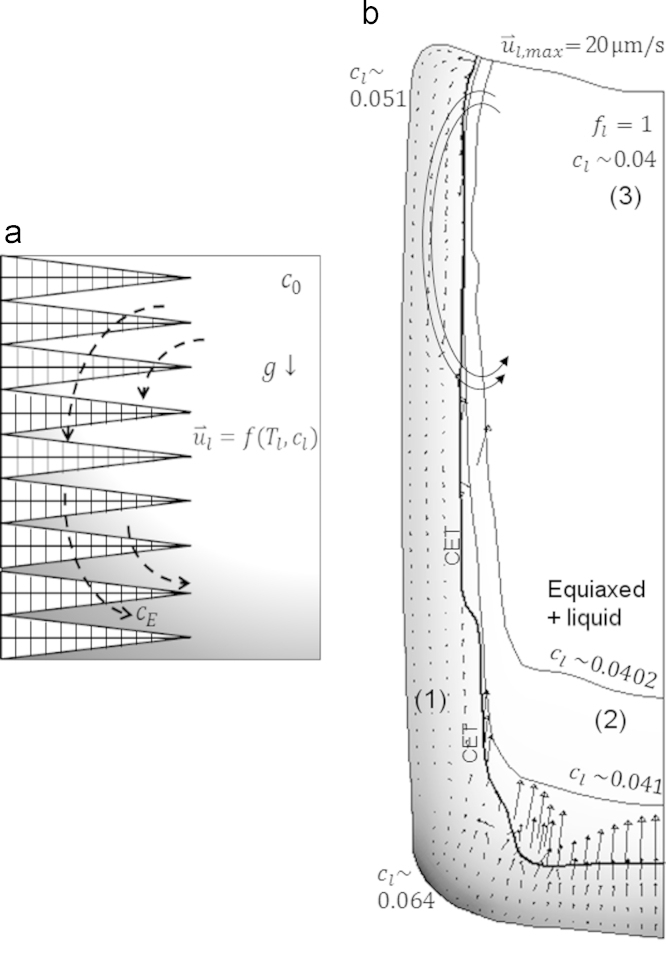
(a) Schematic of solute concentration field during horizontal directional solidification of an alloy with k<1 and (b) the calculated concentration field of liquid during solidification of Al-4 wt.%Cu ingot after 119 s with ${\vec{u}}_{l}$ overlaid.

The formation of macrosegregation is the consequence of the melt flow, crystal sedimentation and their interaction with the solidification. It is convenient to recall the most simplified horizontal directional solidification case of a binary alloy system with denser solute, for example Al-4 wt.%Cu as shown in Fig. 7a. In this case, the solute is rejected from the solidifying dendrites to the interdendritic liquid in the mush and to the extradendritic liquid ahead of the primary dendrite tips. The solute concentration and temperature vary ahead of the growing columnar front. Consequently, liquid metal from the bulk flows downward against the growth direction. This flow pattern affects the melt constitution. Accordingly the upper columnar tips receives higher undercooling compared to the lower ones and a faster growth rate is expected at the top compared to the bottom. In addition, the interdendritic liquid flow washes the solute-rich liquid down forming a positive segregation, particularly close to the bottom.

**Fig. 8:**
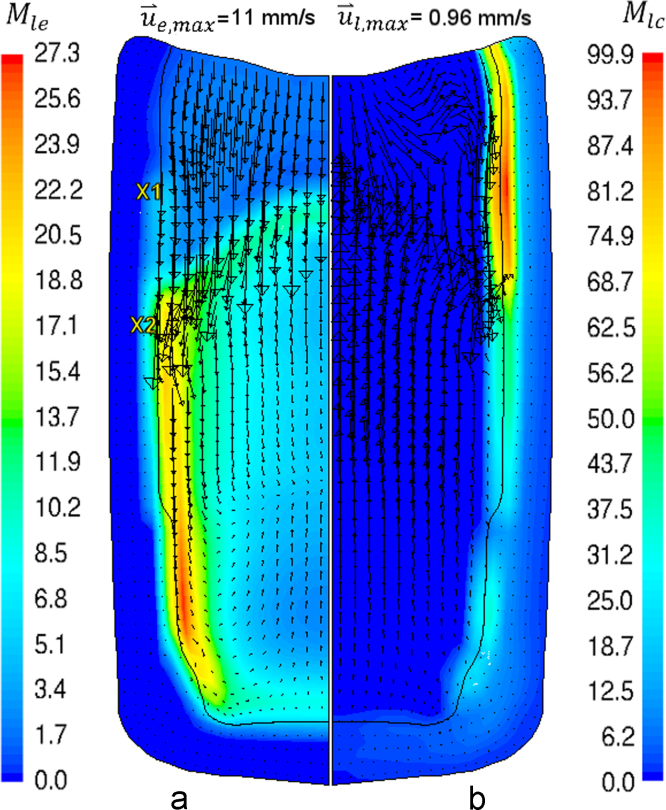
The calculated mass transfer rate $\left[\text{kg}\;m^{-3}\;s^{-1}\right]$ between (a) liquid-equiaxed, with ${\vec{u}}_{e}$ superimposed and (b) liquid-columnar, with ${\vec{u}}_{l}$ superimposed.

The current ingot is cooled from the bottom and at the side walls. A segregation pattern similar to that shown in Fig. 7a can be expected if only columnar dendrites form. Nevertheless, equiaxed crystals nucleate at the mold wall and in the liquid bulk. The sedimentation and the interaction of such crystals with the other phases make the expectation of the segregation pattern quite tricky. The calculated $c_{l}$ at an intermediate solidification stage is shown in Fig. 7b. Three zones can be distinguished based on solute concentration of liquid: (1) the highly enriched columnar mush, particularly at bottom corner, (2) the relatively enriched bottom of the equiaxed basin and (3) the bulk liquid at the top centre of the ingot with $c_{l}\approx c_{0}$. Maximum $c_{l}$, 0.064, occurs at the bottom corner of zone (1) which is characterized by high cooling and solidification rates (Fig. 8b) that increases the rate of solute rejection to liquid. Moreover, the solute rich liquid in this zone is almost stagnant due to the reduced mush permeability. The top of this zone is less segregated ($c_{l}$~0.051) compared to the bottom ($c_{l}$~0.064) due to the relatively stronger interdendritic flow that washes the rejected solute downward, similar to the schematic shown in Fig. 7a. The solute carried by the liquid from the bottom columnar mush and that rejected by the solidification of equiaxed crystals cause enrichment of liquid of the zone (2) in Cu. Because of the very low solidification rate in the bulk liquid, zone (3) encountered no solute enrichment ($c_{l}\;\approx c_{0}$).

### 4.2 Segregation due to equiaxed motion

Further analysis of solidification sequence (Fig. 3) is necessary to explain how macrosegregation forms. As an example, the interaction between phases is explored (119 s, Fig. 3e). The calculated liquid/equiaxed and liquid/columnar mass transfer rates (the source terms, Eqs. (17) and (18)), with respectively superimposed ${\vec{u}}_{e}$ and ${\vec{u}}_{l}$, are shown in Fig. 8.

The equiaxed crystals are depleted in the upper part of the ingot (Fig. 3) due to the continual sedimentation and the reduced nucleation rate in this relatively hot region. Consequently, the columnar tip front advances progressively and converges towards the ingot centerline (Fig. 3f–h). An extreme $M_{lc}$ is calculated at the upper part of c-front as shown in Fig. 8b. At this region, the liquid encountered high negative thermal and solutal buoyancy (acting downward) due to liquid cooling and solute enrichment, respectively. Therefore, liquid settles down at the wall, carrying the rejected solute from the c-front. The simultaneous sedimentation of equiaxed crystals and the rapid growth and convergence of the upper part of c-front leads to the formation of fully columnar top which agrees perfectly with the experiment as-cast structure. This region is characterized by a negative macrosegregation as shown in Fig. 5b.

Opposite to the upper part of ingot, the extradendritic liquid in the equiaxed ‘basin’ formed at the lower half has velocity vectors pointing upwards as shown in Fig. 8b. This can be attributed to the continuous settling of equiaxed crystals. The equiaxed crystals settle down with ${\vec{u}}_{e,\;\max}=11\;\mathrm{mm}/s$ to replace the volume space of the extradendritic liquid (in each control volume $f_{l}+f_{e}+f_{c}=1$ must be fulfilled), pushing the liquid upwards. The floating solute-rich liquid displaced from the equiaxed basin accumulates at the top of the equiaxed basin and gets together with that settling from the upper ingot half. After solidification the solute-rich liquid transforms into eutectic phase, which introduces the positive macrosegregation at the top CET borders as shown in Fig. 5b.

In contrast to upper part of the c-front, the liquid/columnar mass transfer rate is much lower at c-front in the equiaxed basin (Fig. 8b). The extradendritic melt ahead of the c-front is enriched in solute rejected from the solidification of the equiaxed crystals and carried by the liquid from the bottom columnar shell. As a result, columnar tip undercooling decreases leading to reduction in $M_{lc}$ at the c-front. Respectively, the advancement of c-front is decelerated (i.e. the soft blocking mechanism is activated) compared to the upper ingot half. At late stages of solidification, $f_{e}$ increases tremendously, leading to gradual mechanical blocking starting from the bottom c-front. At the end of solidification, CET forms as shown in Fig. 3i. The positive macrosegregation formed at the bottom and at the vertical boundaries of the equiaxed basin can be attributed to almost stagnant solute-rich liquid in this region and the weak outflow of the solute-enriched interdendritic liquid coming from the root of the columnar mush as previously explained in Fig. 7b. The negative macrosegregation at the centre of the equiaxed basin can be attributed to the high $f_{e}$ and the dispersion of the residual extradendritic liquid. Similarly, equiaxed negative segregation zone appeared in typical steel ingots and is known as ‘equiaxed cone’. Flemings [30] attributed the negative macrosegregation in this zone to the divergence of the flow of the residual liquid through it at a late stage of solidification. The formation of some positive segregation islands in the mixed columnar/equiaxed region of the upper ingot half (Fig. 5b) can be attributed to the deflection of the flow streams of liquid at such regions, for example at the markers X1 and X2 shown in Fig. 8a, which increases $M_{le}$ and solute rejection locally.

### 4.3 Tendency to form channel segregation

**Fig. 9:**
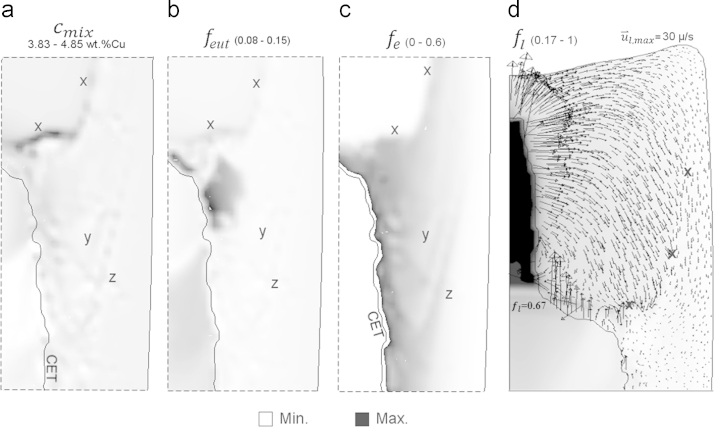
(a) Zoom-in $c_{mix}$ result in the mixed columnar/equiaxed zone as marked in Fig. 6b, and contours of volume fractions of (b) eutectic, (c) equiaxed phase and (d) contour of $f_{l}$ with superimposed ${\vec{u}}_{l}$ at the upper ingot half at 242 s.

It was discussed above that the grid size has minor effect on global CET pattern and solute concentration and the fine grid provides more localized solute distribution that deviates from that averaged by using the coarser grids. However, some segregation features could be resolved by the fine (0.5 mm) grid. By zooming the dashed box shown in Fig. 6b, fine inclined features of positive segregation could be distinguished at the markers x, y, and z as shown in Fig. 9a. Such inclined features were aligned bands of high eutectic and equiaxed volume fractions as shown in Fig. 9b and c. Melt flow in the mushy zone of the upper half of the ingot was explored at earlier stage of solidification. The fraction and the velocity vectors of the extradendritic liquid at 242 s are shown in Fig. 9d (compare with $f_{e}$ and ${\vec{u}}_{e}$ shown in Fig. 3h). It is obvious that the solute-rich liquid encounters an internal resistance in the mixed columnar/equiaxed mush as it comes closer to the ingot surface. In this surface region, the stream lines of liquid adapt themselves and coalesce to follow the more permeable path (marked with ‘x’) as shown in Fig. 9d. The solute-rich liquid streams are gathered in an inclined channel which pumps the liquid into a ‘lagoon’ with $f_{l}$~0.67 formed below the boundary of the c-front. The solute-rich liquid ‘lagoon’ and channels solidify in a later stage forming eutectic (Fig. 9b) with positive segregation. Similarly, inclined features marked ‘y’ and ‘z’ are also evident as shown in Fig. 9a–c but with weaker liquid flow.

**Fig. 10:**
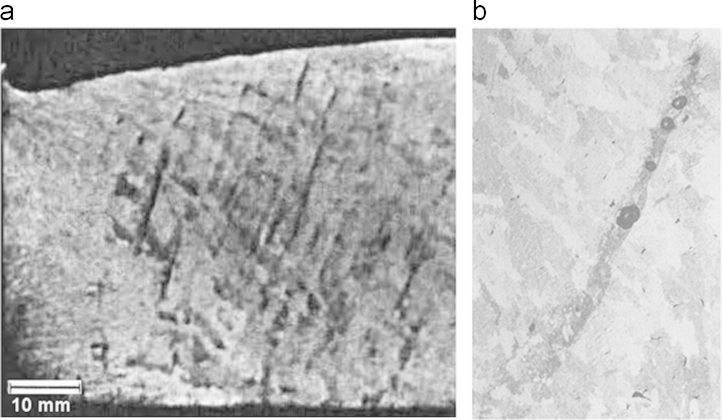
Channel segregation of horizontally solidified (a) Al-5.5 wt.%Cu [31] and (b) Al-20 wt.%Cu [30].

The abovementioned flow features are thought to form the channel segregation. Several experimental evidence revealed the tendency of Al-Cu alloys to form channel segregation as shown in Fig. 10. Flemings suggested that the stream of the solute-rich interdendritic liquid may coalesce and cause dendrite remelting which leads to the formation of inclined channel segregation [30]. Mori and Ōgi observed many channels in the Al-5.5 wt.% Cu sample [31]. These channels end into the bulk liquid at the lower part of the solidification interface analogous to the simulation results. The channel segregation is not detected in the current experiment, probably due to the low resolution of the measuring method or the small size of the ingot. In addition, the intricate 3D non-planar nature of the channels might require more specific characterization procedure using different cut plains. Further numerical and experimental investigations for this phenomenon are intended in the future work.

## 5 Conclusion

The 5-phase mixed columnar-equiaxed solidification model succeeded to qualitatively predict the as-cast structure including CET and the macrosegregation pattern of an Al-4 wt.%Cu ingot.

The formation of CET is governed by the competitive growth between the columnar and equiaxed phases and the nucleation and sedimentation of equiaxed crystals. Solute rejected from the growing equiaxed dendrites ahead of the columnar primary tip front decelerates its advancement (soft blocking mechanism). Sedimentation and growth of equiaxed crystals stop the growth of the columnar primary tip front if $f_{e}$>0.49 ahead of the front is fulfilled (hard blocking mechanism).

Regions of positive and negative macrosegregation are numerically predicted in agreement with experiment. The accumulation of the solute-rich liquid driven by the thermosolutal buoyancy is the main origin of the positive macrosegregation. Sedimentation and growth of equiaxed crystals form the equiaxed basin at ingot core with negative macrosegregation. Rapid growth of columnar grains forms pure columnar region in the upper part of the ingot with negative macrosegregation.

Despite the grid dependence, the qualitative results of the structure, CET and macrosegregation are not significantly affected by grid size. In contrast to the coarse grid, the fine grid provides much localized results. Furthermore, some inclined segregation bands appear in the mixed structure zone using the fine grid (0.5 mm). Such features are supposed to be channel segregation. Further experimental investigations to detect segregation channels are intended in the outlook.

## Figures and Tables

**Table 1:** Conservation and transport equations, and some of source and exchange terms.

I. Conservation and transport equations
Mass:
(1) $$\frac{\partial }{\partial t}\left(f_{l}\rho_{l}\right)+\;\nabla \cdot \left(f_{l}\rho_{l}{\vec{u}}_{l}\right)=-M_{le}-M_{lc}$$
(2) $$\frac{\partial }{\partial t}\left(f_{e}\rho_{e}\right)+\;\nabla \cdot \left(f_{e}\rho_{e}{\vec{u}}_{e}\right)=M_{le}$$
(3) $$\frac{\partial }{\partial t}\left(f_{c}\rho_{c}\right)+\;\nabla \cdot \left(f_{c}\rho_{c}{\vec{u}}_{c}\right)=M_{lc}$$
(4) $$\frac{\partial }{\partial t}\left(f_{s}^{e}\rho_{e}\right)+\nabla \cdot \left(f_{s}^{e}\rho_{e}{\vec{u}}_{e}\right)=M_{ds}^{e}$$
(5) $$\frac{\partial }{\partial t}\left(f_{s}^{c}\rho_{c}\right)+\nabla \cdot \left(f_{s}^{c}\rho_{c}{\vec{u}}_{c}\right)=M_{ds}^{c}$$
Species:
(6) $$\frac{\partial }{\partial t}\left(f_{l}\rho_{l}c_{l}\right)\;+\;\nabla \cdot \left(f_{l}\rho_{l}{\vec{u}}_{l}c_{l}\right)=-C_{le}-C_{lc}$$
(7) $$\frac{\partial }{\partial t}\left(f_{e}\rho_{e}c_{e}\right)+\nabla \cdot \left(f_{e}\rho_{e}{\vec{u}}_{e}c_{e}\right)=C_{le}$$
(8) $$\frac{\partial }{\partial t}\left(f_{c}\rho_{c}c_{c}\right)+\nabla \cdot \left(f_{c}\rho_{c}{\vec{u}}_{c}c_{c}\right)=C_{lc}$$
(9) $$\frac{\partial }{\partial t}\left(f_{s}^{e}\rho_{e}c_{s}^{e}\right)+\nabla \cdot \left(f_{s}^{e}\rho_{e}{\vec{u}}_{e}c_{s}^{e}\right)=C_{ds}^{e}$$
(10) $$\frac{\partial }{\partial t}\left(f_{s}^{c}\rho_{c}c_{s}^{c}\right)+\nabla \cdot \left(f_{s}^{c}\rho_{c}{\vec{u}}_{c}c_{s}^{c}\right)=C_{ds}^{c}$$
Momentum:
(11) $$\frac{\partial }{\partial t}\left(f_{l}\rho_{l}{\vec{u}}_{l}\right)+\;\nabla \cdot \left(f_{l}\rho_{l}{\vec{u}}_{l}⊗{\vec{u}}_{l}\right)=-f_{l}\nabla p+\nabla \cdot {\bar{\bar{\tau }}}_{l}+f_{l}\rho_{l}g-{\vec{u}}_{le}^{p}-{\vec{u}}_{lc}^{p}-{\vec{U}}_{le}^{d}-{\vec{u}}_{lc}^{d}$$
(12) $$\frac{\partial }{\partial t}\left(f_{e}\rho_{e}{\vec{u}}_{e}\right)+\;\nabla \cdot \left(f_{e}\rho_{e}{\vec{u}}_{e}⊗{\vec{u}}_{e}\right)=-f_{e}\nabla p+\nabla \cdot {\bar{\bar{\tau }}}_{e}+f_{e}\rho_{e}g+{\vec{U}}_{le}^{p}+{\vec{U}}_{le}^{d}$$
Enthalpy:
(13) $$\frac{\partial }{\partial t}\left(f_{l}\rho_{l}h_{l}\right)+\nabla \cdot \left(f_{l}\rho_{l}{\vec{u}}_{l}h_{l}\right)=\nabla \cdot \left(k_{l}\nabla \cdot T_{l}\right)+Q_{el}^{p}+Q_{cl}^{p}+Q_{el}^{d}+Q_{cl}^{d}$$
(14) $$\frac{\partial }{\partial t}\left(f_{e}\rho_{e}h_{e}\right)+\nabla \cdot \left(f_{e}\rho_{e}{\vec{u}}_{e}h_{e}\right)=\nabla \cdot \left(k_{e}\nabla \cdot T_{e}\right)-Q_{el}^{p}-Q_{el}^{d}-Q_{ce}^{d}$$
(15) $$\frac{\partial }{\partial t}\left(f_{c}\rho_{c}h_{c}\right)=\nabla \cdot \left(k_{c}\nabla \cdot T_{c}\right)-Q_{cl}^{p}-Q_{cl}^{d}+Q_{ce}^{d}$$
Equiaxed number density:
(16) $$\frac{\partial }{\partial t}n+\nabla \cdot ({\vec{u}}_{e}\cdot n)=N$$
II. Source terms for mass conservation equations
(17) $$M_{le}=v_{env}^{e}\cdot S_{env,M}^{e}\cdot \rho_{e}$$
(18) $$M_{lc}=\{\begin{array}{ll} v_{env}^{c}\;S_{env,M}^{c}\;\rho_{c}, & Trunk\;cell\\ M_{tip^{׳}}^{c}, & Tip\;cell:\;\;l\leq R_{tip^{׳}}^{c}\\ v_{env}^{c}\;S_{env,M}^{c}\;\rho_{c}+M_{tip^{׳}}^{c}, & Tip\;cell:\;l>R_{tip^{׳}}^{c} \end{array}$$
(19) $$M_{ds}^{e}=\{\begin{array}{ll} M_{le}, & Globular\\ v_{sd}^{e}\;S_{s}^{e}\;\rho_{e}, & Dendritic \end{array}$$
(20) $$M_{ds}^{c}=\{\begin{array}{ll} M_{lc}, & Cellular\\ v_{sd}^{c}\;S_{s}^{c}\;\rho_{c},\; & Dendritic,\;Trunk\;cell\\ M_{lc}, & Dendritic,Tip\;cell:\;\;l\leq R_{tip^{׳}}^{c}\\ v_{sd}^{c}\;S_{s}^{c}\;\rho_{c}+M_{tip^{׳}}^{c}, & Dendritic,Tip\;cell:\;\;l>R_{tip^{׳}}^{c} \end{array}$$
$$\begin{array}{l} \mathrm{Where},M_{tip^{׳}}^{c}=v_{tip׳}^{c}\;\left(\pi \;n_{c}R_{tip^{׳}}^{2}\right)\;\rho_{c}\;f_{l}\\ v_{env}^{e}=\;\max(v_{glob}^{e},v_{env,\;M}^{e})\;\mathrm{and}\\ v_{env}^{c}=\;\max(v_{cell}^{c},v_{env,\;M}^{c}) \end{array}$$
III. Source terms for species conservation equations
(21) $$C_{le}=\{\begin{array}{ll} c_{s}^{⁎}\cdot M_{le} & globular\\ c_{s}^{⁎}\cdot M_{le}+C_{le}^{D}\; & dendritic \end{array}$$
(22) $$C_{lc}=\{\begin{array}{ll} c_{s}^{⁎}\cdot M_{lc} & cellular\\ c_{s}^{⁎}\cdot M_{le}+C_{lc}^{D} & dendritic \end{array}$$
(23) $$C_{ds}^{e}=c_{s}^{⁎}\cdot M_{ds}^{e}\;\mathrm{only}\;\mathrm{dendritic}$$
(24) $$C_{ds}^{c}=c_{s}^{⁎}\cdot M_{ds}^{c}\;\;\mathrm{only}\;\mathrm{dendritic}$$
IV. Auxiliary correlations:
(25) $$\Delta T=(T_{f}+mc_{l})-T$$
(26) $$c_{mix}=\frac{c_{l}\rho_{l}f_{l}+c_{e}\rho_{e}f_{e}+c_{c}\rho_{c}f_{c}}{\rho_{l}f_{l}+\rho_{e}f_{e}+\rho_{c}f_{c}}$$

**Table 2:** Thermophysical properties and modeling parameters of Al-4.0 wt.%Cu alloy.

**Thermophysical properties**	**Nucleation parameters**
$c_{0}$=4.0 wt.% Cu, $c_{E}$=33.2 wt.% Cu	$n_{max}$=1.48×10^11^ m^−3^$\Delta T_{\sigma }$=28.84 K
Liquidus temperature=922.15 K	$\Delta T_{max}$=10.17 K

Eutectic temperature=821.35 K	**Morphological parameters:**
m=−3.44 K/wt.%, k=0.145	Equiaxed dendrite:
$D_{l}$=5.0×10^−09^ m^2^/s, $D_{s}$=8.0×10^−13^ m^2^/s	Shape factor=0.48, sphericity=0.4
Latent heat of fusion=389.32 kJ/kg	
Thermal capacity=1100 J/kg·K	Columnar dendrite:
Thermal conductivity=87 W/m·K	Shape factor=0.80, trunk circularity=0.5
Liquid viscosity=1.28×10^−3^ kg/m·s	λ_1_=500 μm, λ_2_=100 μm

Thermal expansion coefficient=10^−4^ K^−1^	**Tip growth parameters for KGT model:**
Solutal expansion coefficient=−0.92 wt.%^−1^	K_1_=2.0813×10^−5^, K_2_=2.798×10^−5^
